# Transfer of metastatic traits via miR‐200c in extracellular vesicles derived from colorectal cancer stem cells is inhibited by atractylenolide I

**DOI:** 10.1002/ctm2.139

**Published:** 2020-08-12

**Authors:** Dongxin Tang, Xiaofen Xu, Jialiang Ying, Tian Xie, Gang Cao

**Affiliations:** ^1^ School of Pharmacy Zhe jiang Chinese Medical University Hangzhou China; ^2^ First Affiliated Hospital Guizhou University of Traditional Chinese Medicine Guiyang China; ^3^ Innovative Institute of Chinese Medicine and Pharmacy Chengdu University of Traditional Chinese Medicine Chengdu China

**Keywords:** atractylenolide I, extracellular vesicles, metastasis, PI3K/Akt/mTOR, stemness

## Abstract

Cancer stem cells (CSCs) are important factors contributing to tumorigenesis. We examined whether CSCs isolated from colorectal cancer (CRC) cells possess metastatic properties that can be transferred to non‐CSCs via the delivery of miR‐200c enclosed in extracellular vesicles (EVs). The inhibitory effect of atractylenolide I (ATL‐1), a traditional Chinese medicinal compound, on miR‐200c activity and metastatic transfer was investigated. EVs were isolated from colorectal CSCs. The expression of miR‐200c was evaluated in CSCs and CSC‐derived EVs, and horizontal transfer of metastatic properties via EVs to non‐CSCs was investigated in terms of cell behavior and phosphatidylinositol‐4,5‐bisphosphate 3‐kinase (PI3K)/protein kinase B (Akt)/mammalian target of rapamycin (mTOR) signaling. CSCs isolated from metastatic CRC cells exhibited higher levels of miR‐200c than those in nonmetastatic CRC cells. Overexpression of miR‐200c in CSCs enhanced metastatic potential by promoting proliferation and inhibiting apoptosis, in turn leading to the release of EVs carrying an excess of miR‐200c. Non‐CSCs co‐cultured with miR‐200c‐containing EVs exhibited enhanced invasion and stemness maintenance associated with PI3K/Akt/mTOR activation, demonstrating successful metastatic transfer via EV delivery. Furthermore, ATL‐1 impaired the EV‐mediated transfer of metastatic properties by suppressing miR‐200c activity and disrupting EV uptake by non‐CSCs. EVs are critical signal transducers that facilitate intercellular communication and exchange of metastatic properties, which can be controlled by ATL‐1. The findings are useful in the development of microRNA‐based anticancer strategies by targeting EV‐mediated activity, especially using natural compounds.

## Graphical abstract

A key factor that contributes to colorectal tumour metastasis is the production of a large number of CSCs in the tumour microenvironment. These CSCs secrete EVs that act as carrier vehicles to transport miR‐200c, which regulates stemness maintenance in CRC cells and promotes CRC metastasis. Horizontal transfer of miR‐200c‐mediated properties is facilitated via the co‐culture of CRC cells and miR‐200c‐containing EVs, and the uptake of EVs by CRC cells results in increased metastatic potential, enhanced stem‐like traits, and activation of PI3K/AKT/mTOR signalling. ATL‐1 suppresses the activity of CSCs while inhibiting the uptake of EVs by CRC cells to prevent metastasis.

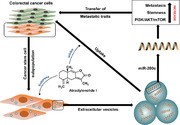

**Abbreviations**
Akt: protein kinase B
ANOVA: analysis of variance
ATL: atractylenolide
CRC: colorectal cancer
CSC: cancer stem cell
EV: extracellular vesicle
FBS: fetal bovine serum
FITC: fluorescein isothiocyanate
miR: microRNA
mTOR: mammalian target of rapamycin
MTT: 3‐(4,5‐dimethylthiazol‐2‐yl)‐2,5‐diphenyltetrazolium bromide
OCT‐4: octamer‐binding transcription factor 4
PBS: phosphate‐buffered saline
PI3K: phosphatidylinositol‐4,5‐bisphosphate 3‐kinase
SD: standard deviation
SOX‐9: sex‐determining region Y‐box
TEM: transmission electron microscopy

## 1 INTRODUCTION

Colorectal cancer (CRC) is one of the most prevalent cancers worldwide, with an estimated 97 000 new cases and 50 000 deaths in 2018 in the United States alone.^1^ Most CRC‐related deaths are caused by metastasis of malignant tumours,^2^ and research on CRC treatment has thus focused on controlling the occurrence of metastasis. The factors that affect CRC metastasis are manifold, and it is challenging to gain a comprehensive view of the mechanisms involved within. MicroRNAs (miRs) have been a recent highlight in diverse areas of medical research, including targeted cancer therapy, because of their effects on a variety of biological responses, such as oncogenesis and tumor metastasis. The identification of specific miRs as oncomiRs or tumor suppressors has advanced our knowledge of cancer development and contributed to the emergence of miR‐based therapeutic schemes. For example, targeting the oncogenic miR‐155 delayed tumor growth,^3^ and a deliverable therapeutic formulation against lung cancer has been developed based on the tumor suppressor miR‐34.^4^ Interestingly, miR‐200c has been identified as both an oncomiR^5^ and a tumor suppressor^6^ in CRC. With these contrasting reports, it is necessary to further investigate the role of miR‐200c in CRC development and metastasis.

The metastatic properties of miR‐200c have been demonstrated to be transferrable via extracellular vesicles (EVs) as carriers between tumor cells with different metastatic abilities.^7^ EVs are spherical particles that are categorized based on their size and origin. For example, those with diameters of 50‐150 nm are known as exosomes and originate from endosomes, whereas those with diameters of 50 nm to 1 μm are known as microvesicles (or oncosomes in cancer cells), originating from the plasma membrane.^8^ EVs derived from cancer stem cells (CSCs) reportedly mediate the crosstalk between cells via the horizontal transfer of tumorigenic factors between cells,^9^ such as oncogenes and proteins. Although CSCs form only a subset of the cancer cell population, they are believed to be a key determinant of tumorigenesis and play important roles in regulating the tumor environment and metastasis. The maintenance of stem‐like properties in CSCs is critical in promoting disease onset and thus, studies have focused on therapeutic means of disrupting or impairing the maintenance of stemness.^10^, ^11^ Whether the pro‐tumorigenic potential of CSCs, such as metastatic and stem‐like traits, can be conferred to nonmetastatic CRC cells via the transfer of miR‐containing EVs remains thus far unknown.

Aside from conventional anticancer schemes such as surgical intervention and chemotherapy, natural herbal and plant‐based compounds have gained attention as adjuvant or complementary therapies in cancer treatment. Baizhu, or Atractylodes macrocephala Koidz, is a dry perennial rhizome in the Asteraceae family and has been applied as a typical component of many traditional Chinese medical formulations.^12^, ^13^ Among the components of baizhu, three forms of atractylenolides (ATLs) (I, II, and III) have been identified to exert pharmacological properties, and ATL‐1 is the main active ingredient that has exhibited therapeutic effects against CRC.^14^, ^15^, ^16^, ^17^ Despite this, studies on ATL‐1 in contemporary cancer treatment are scarce, as are those on its effects on CSCs and the dynamics of miR‐carrying EVs.

We are interested in investigating whether the metastatic potential of CRC cells can be amplified when they are cultured with CSC‐derived EVs carrying miR‐200c, which is presumed to be oncogenic. We also examined whether ATL‐1 possesses the ability to suppress CRC metastasis by exploring its effect on the transfer of miR‐200c by EVs and provide a speculation on the mechanisms involved in its antitumorigenic action.

## 2 MATERIALS AND METHODS

### 2.1 Cell culture, transfection, and treatment

Two human CRC cell lines (high‐metastasis LoVo cells and low‐metastasis HT29 cells) were obtained from the cell bank of the Chinese Academy of Sciences Shanghai Branch (Shanghai, China). LoVo cells were cultured in F12K medium (21127‐022, Gibco, Waltham, MA) containing 10% fetal bovine serum (FBS; 10270‐106, Gibco) and HT29 cells were cultured in McCoy's 5A medium (16600‐082, Gibco) containing 10% FBS at 37°C in an atmosphere containing 5% CO_2_. To isolate CSCs from LoVo or HT29 cells (denoted as LoVo‐CSCs and HT29‐CSCs, respectively), four Eppendorf tubes were prepared and 100 μL of cell suspension (1 × 10^8^ cells/mL) were added to each tube. Then, 2 μL of CD44 antibody (85‐11‐0441‐82, eBioscience, Waltham, MA) was added to tube 1, 2 μL of epithelial cell adhesion molecule (EpCAM) antibody (12‐5791‐81, eBioscience) was added to tube 2, CD44 and EpCAM antibody (2 μL of each) were added to tube 3, and no antibody was added to tube 4 (empty label). After 45 min of incubation at 4°C, the cells were subjected to flow cytometric sorting. The isolated CSCs were stored in sterile Eppendorf tubes until use. To overexpress or silence miR‐200c, mimics and inhibitors (GenePharma, Shanghai, China) were transfected into CRC cells or colorectal CSCs. Transfection was performed by incubating cells with miR‐200c mimics or inhibitors at 50 nM for 48 h. For experiments involving ATL‐1 treatment, 20 mg of ATL‐1 (purity ≥98% HPLC; batch no: DST170606‐014, Dsiter, Chengdu, China) was prepared in 500 μL of dimethyl sulfoxide to prepare a stock solution with a concentration of approximately 175 mM. The stock solution was then diluted in culture medium and administered at 200 μM for 48 h. Phosphate‐buffered saline (PBS) was used as a control.

### 2.2 Isolation and characterization of CSC‐derived EVs

EVs were isolated from nontreated or transfected LoVo‐CSCs or HT29‐CSCs using the ExoQuick‐TC Exosome Precipitation Solution for Culture Media, Spinal Fluid and Urine (EXOTC50A‐1‐SBI, System Biosciences, Palo Alto, CA). The cells were cultured in FBS‐free low‐glucose Dulbecco's modified Eagle medium for 48 h, after which the medium was collected and centrifuged at 500 × *g* for 10 min. The supernatant was collected and centrifuged at 2000 × *g* for 20 min, and the supernatant was collected again and ultracentrifuged at 100 000 × *g* for 70 min. The precipitate was resuspended in 20 mL of PBS and ultracentrifuged at 100 000 × *g* for 70 min, after which the precipitate was resuspended in PBS at a ratio of 1:20. The mixture was centrifuged at 2000 × *g* for 20 min, and the supernatant was subjected to sucrose density gradient purification of EVs. After the gradient was ultracentrifuged at 100 000 × *g* for 70 min, the EV fraction (∼40% sucrose) was carefully collected using a long pipette tip. The collected fraction was ultracentrifuged at 100 000 × *g* for 70 min, and the resulting precipitate containing isolated EVs was collected. All subsequent experiments involving co‐culture with EVs (except for PKH labeling) were performed with 100 μg/mL EVs for 48 h.

### 2.3 Transmission electron microscopy

Transmission electron microscopy (TEM) was performed to identify the isolated EVs. The EVs were fixed with 2% glutaraldehyde (in 0.1 M PBS, pH 7.4), and the fixed EVs were added dropwise to a treated nickel mesh for 30 min. After the mesh was washed with PBS, 1% glutaraldehyde was added dropwise and incubated for 5 min, after which the mesh was washed several times with double‐distilled water. Then, filtered 4% uranyl acetate was added to the sample dropwise and incubated for 5 min. Excess liquid was blotted with filter paper and the sample was dried. The morphology of the EVs was observed using TEM.

### 2.4 3‐(4,5‐Dimethylthiazol‐2‐yl)‐2,5‐diphenyltetrazolium bromide assay

CRC cells or colorectal CSCs in the logarithmic growth phase were collected for 3‐(4,5‐dimethylthiazol‐2‐yl)‐2,5‐diphenyltetrazolium bromide (MTT) assay. The cells were seeded in 96‐well plates at 5 × 10^3^ cells/well and cultured overnight at 37°C. The cells were subjected to transfection or ATL‐1 treatment as described in Section 2.2, if applicable. After 24, 48, or 72 h of culture, 10 μL of 5 mg/mL MTT reagent (PAB180013, Bioswamp, Wuhan, China) was added to each well and the cells were further cultured for 4 h. Then, the MTT solution was removed and 150 μL of dimethyl sulfoxide was added to each well. The plate was gently shaken for 10 min and the absorbance of the wells was measured using a plate reader at 490 nm.

### 2.5 Transwell assay of cell migration and invasion

Transwell chambers (Corning Inc., Corning, NY) were placed in the wells of a 24‐well plate and immersed in PBS for 5 min before the experiment. After cells were subjected to 100 μg/mL EV and/or 200 μM ATL‐1 treatment for 48 h, they were cultured in FBS‐free medium for 24 h. For the migration assay,^18^ the cells were trypsinised, resuspended in 1% FBS, and seeded into the upper Transwell chambers at 1 × 10^5^ cells/mL (0.5 mL/well). In the bottom Transwell chambers, 0.75 mL of medium containing 10% FBS was added in each well. The plates were incubated at 37°C for 48 h, and the cells were fixed with 1 mL of 4% paraformaldehyde in each well for 10 min at room temperature. The fixative was removed and the cells were washed once with PBS. Then, 1 mL of 0.5% crystal violet solution (PAB180004, Bioswamp) was added to each well, and after 30 min of staining, the cells were washed three times with PBS. Cells that did not migrate were removed using a cotton swab, and migrated cells were observed and counted at 200× using an inverted microscope (DM IL LED, Leica Microsystems, Wetzlar, Germany). The invasion assay was performed following the same procedure, except that each chamber was coated with 80 μL of Matrigel (354230, BD Biosciences, Franklin Lakes, NJ) at 37°C for 30 min prior to cell seeding.

### 2.6 Flow cytometry detection of stem cell markers and apoptosis

The purity of isolated CSCs was evaluated by flow cytometry using CD44 and EpCAM.^19^ CSCs (1 × 10^6^) were suspended in 100 μL of flow cytometry buffer in an Eppendorf tube and 2 μL of fluorescein isothiocyanate (FITC)‐conjugated CD44 or phycoerythrin‐conjugated EpCAM antibody was added to each tube. The tubes were incubated at 4°C for 30 in the absence of light. Then, the cells were washed with 2 mL of buffer and centrifuged at 300 × *g* at 4°C for 5 min, and the supernatant was removed. The cells were resuspended in 400 μL of buffer and subjected to flow cytometry using a CytoFLEX S apparatus (Beckman Coulter, Brea, CA). Data were analyzed using NovoExpress software (ACEA Biosciences, Inc., San Diego, CA). For apoptosis, flow cytometry was performed using a FITC‐AnnexinV Apoptosis Detection Kit (556547, BD Biosciences), 48 h after CRC cells or colorectal CSCs were treated with 200 μM ATL‐1 or subjected to transfection. Trypsinised cells were centrifuged at 1000 × *g* for 5 min and the supernatant was removed. Approximately 1 × 10^6^ cells were resuspended in PBS and centrifuged at 1000 × *g* at 4°C for 5 min. The previous step was performed three times and the cells were resuspended in 200 μL of binding buffer. Thereafter, 10 μL of annexin V‐FITC and 10 μL of propidium iodide were added to the cells. After 30 min of incubation at 4°C in the dark, 300 μL of binding buffer was added and the cells were subjected to flow cytometry. The data were analyzed using NovoExpress software.

### 2.7 Quantitative reverse transcription polymerase chain reaction

Stem‐loop quantitative reverse transcription polymerase chain reaction was performed to quantify the expression of miR‐200c in CRC cells, colorectal CSCs, and CSC‐derived EVs. After 48 h of culture with 100 μg/mL EVs and/or 200 μM ATL‐1, RNA was extracted using TRIzol (15596026, Ambion Inc., Foster City, CA) according to the manufacturer's instructions, and reverse transcription of the extracted RNA into cDNA was carried out using the Advantage RT‐for‐PCR kit (639505, TaKaRa, Dalian, China). Quantitative polymerase chain reaction was conducted using the SYBR Green PCR reagent kit (KM4101, KAPABiosystems) with the primers listed in Table 1. PCR proceeded as follows: initial denaturation for 3 min at 95°C; 39 cycles of denaturation for 5 s at 95°C, annealing for 10 s at 56°C, and extension for 25 s at 72°C; and final extension for 5 s at 65°C and 50 s at 95°C. Data were acquired using qbase plus software and analyzed by the 2^−ΔΔ^ *^Ct^* method.

**TABLE 1:** List of primers for quantitative reverse transcription polymerase chain reaction

Primer name	Sequence
miR‐200c stem‐loop	CTCAACTGGTGTCGTGGAGTCGGCAATTCAGTTGAGTCCATCAT
miR‐200c forward	GGGTAATACTGCCGGGTA
miR‐200c reverse	AACTGGTGTCGTGGAGTCGGC
U6 forward	CTCGCTTCGGCAGCACA
U6 reverse	AACGCTTCACGAATTTGCGT

### 2.8 Western blot

To confirm that EV isolation was carried out successfully, western blot of exosomal markers (CD63, CD81, and TSG101) was performed in CSCs, CSC‐derived EVs, and the lysates of cells from which the EVs were isolated. Proteins related to stemness maintenance and phosphatidylinositol‐4,5‐bisphosphate 3‐kinase (PI3K)/protein kinase B (Akt)/mammalian target of rapamycin (mTOR) signaling were also evaluated by western blot. After 48 h of culture with 100 μg/mL EVs and/or 200 μM ATL‐1, proteins were extracted from cells or EVs. Radioimmunoprecipitation assay buffer (PAB180006, Bioswamp) containing protease and phosphatase inhibitors was added to lyse the cells at 4°C. The lysates were transferred to an Eppendorf tube, heated for 10 min at 95°C, and centrifuged at 12 000 × *g* for 10 min. The protein content in the supernatant was quantified using a bicinchoninic acid assay kit (PAB180007, Bioswamp). For western blot, 20 μg of protein was subjected to sodium dodecyl sulfate‐polyacrylamide gel electrophoresis. The electrophoresed proteins were transferred to a polyvinylidene fluoride membrane (IPVH00010, Millipore, Burlington, MA) and blocked using 5% skim milk for 2 h at room temperature. Thereafter, the membranes were incubated overnight at 4°C with rabbit primary antibodies against the following proteins: octamer‐binding transcription factor 4 (OCT‐4, 1:1000, AB18976, Abcam, Cambridge, UK), sex‐determining region Y‐box (SOX‐9, 1:1000, AB185966, Abcam), Nanog (1:1000, AB106465, Abcam), CD63 (1:1000, PAB33929, Bioswamp), CD81 (1:1000, PAB33928, Bioswamp), TSG101 (1:1000, PAB32949, Bioswamp), PI3K (1:1000, AB191606, Abcam), phospho‐PI3K (p‐PI3K, 1:1000, AB182651, Abcam), Akt (1:1000, AB8805, Abcam), p‐Akt (1:1000, AB38449, Abcam), mTOR (1:2000, AB32028, Abcam), p‐mTOR (1:1000, AB84400, Abcam), and GAPDH (1:5000, PAB36264, Bioswamp). After three washes with PBS/Tween 20 for 5 min each, the membranes were incubated with goat antirabbit IgG secondary antibody (1:10000, SAB43711, Bioswamp) for 1 h at room temperature and washed again three times with PBS for 5 min each. For visualization of the proteins, the membranes were incubated with an enhanced chemiluminescence agent (WBKLS0010, Millipore) in the dark and protein band gray values were analyzed using an automatic scanner (Tanon‐5200, Tanon, Shanghai, China). The data are presented as the ratio to the gray value of the control group (taken as a value of 1) in arbitrary units (a.u.).

### 2.9 Evaluation of EV uptake via PKH67 fluorescence labeling

EVs were labeled following the instructions of the PKH67 Green Fluorescent Cell Linker Midi Kit (MIDI67‐1KT, Sigma‐Aldrich, St. Louis, MO). Isolated EVs were mixed with 1 mL of diluent and 4 μL of PKH67 solution (both provided in the kit) and incubated for 4 min. Then, 2 mL of 0.5% bovine serum albumin in PBS was added for quenching. The labeled EVs were ultracentrifuged twice at 100 000 × *g* at 4°C for 70 min and resuspended in 100 μL of PBS. To examine EV uptake, LoVo or HT29 cells were seeded in a 24‐well plate at 1 × 10^4^ cells/well and incubated at 37°C for 12 h in an atmosphere containing 5% CO_2_. Then, 10 μg of PKH67‐labeled EVs were added to each well and the plate was further incubated at 37°C in 5% CO_2_ for 2 or 12 h. The cells were fixed for 20 min with 4% paraformaldehyde and the nuclei were stained with Hoechst 33258, after which they were observed under a Leica DM IL LED fluorescence microscope using a 400× objective lens for EV uptake. The microscopy imaging parameters were set at initial acquisition and were kept constant between acquisitions. Green fluorescence represents PKH67 labeling of EVs.

### 2.10 Statistical analysis

Statistical analysis was performed using OriginPro 8. The data are expressed as the mean ± standard deviation (SD) of three technical replicates (n = 3). Differences between two groups were compared using one‐sample *t*‐test. One‐way analysis of variance (ANOVA) followed by Tukey's post hoc test was conducted for comparisons between multiple groups (more than two). *P* < .05 indicates statistical significance.

## 3 RESULTS

### 3.1 Effect of ATL‐1 on the behavior and properties of colorectal CSCs

**FIGURE 1:**
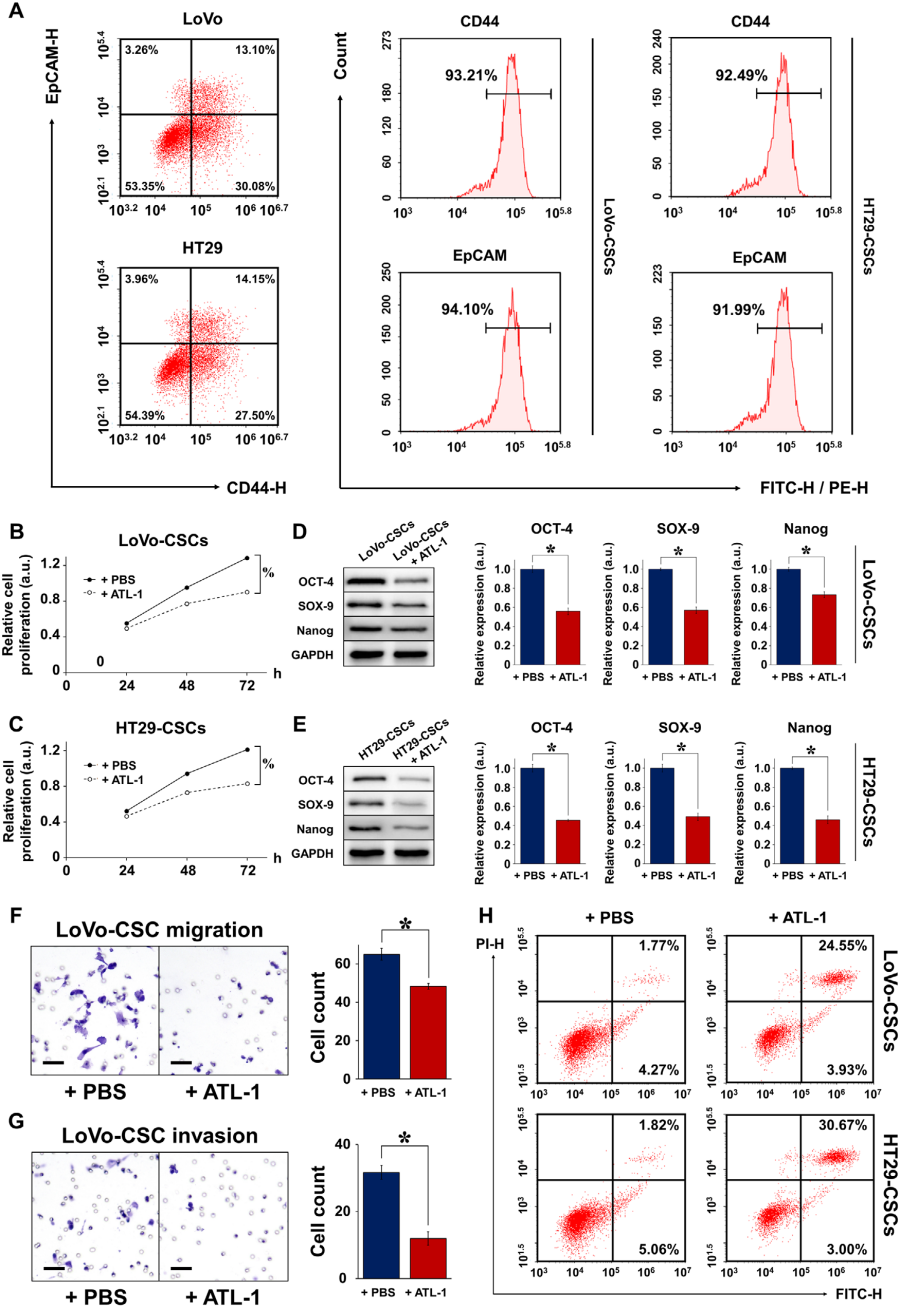
Characterization of colorectal CSCs isolated from LoVo and HT29 cells. A, Flow cytometric sorting of CSCs using CD44 and EpCAM as markers. The percentage of parental LoVo and HT29 cells expressing both CD44 and EpCAM was approximately 15%, representing the proportion of CSCs. CSCs isolated from parental LoVo and HT29 cells (denoted as LoVo‐CSCs and HT29‐CSCs, respectively) exhibited high expression of both markers (>90%). Relative proliferation of (B) LoVo‐CSCs and (C) HT29‐CSCs was inhibited by ATL‐1 at 200 μM for up to 72 h, compared to that of control (+PBS) CSCs. The protein expression of stemness markers OCT‐4, SOX‐9, and Nanog, relative to GAPDH, in (D) LoVo‐CSCs and (E) HT29‐CSCs was downregulated by ATL‐1 at 200 μM after 48 h of culture, compared to that of control (+PBS) CSCs. Transwell assay of the (F) migration and (G) invasion of LoVo‐CSCs demonstrated decreased cell counts in both cases when ATL‐1 was administered at 200 μM, compared to those of control (+PBS) CSCs. H, The percentage of apoptotic LoVo‐CSCs and HT29‐CSCs was elevated in the presence of ATL‐1 at 200 μM, compared to that of control (+PBS) CSCs. The data represent the mean ± SD of three independent technical replicates (*t*‐test); ^*^ *P* < .05; % *P* < .05 at 72 h

Colorectal CSCs were successfully isolated from LoVo and HT29 cells, as evidenced by the flow cytometric profiles of CD44 and EpCAM (Figures 1A and S1), which are markers of CSCs.^15^ We first evaluated the influence of 200 μM ATL‐1 on the behavior of colorectal CSCs. Figures 1B and 1C demonstrate that in CSCs isolated from both LoVo (high metastatic potential) and HT29 (low metastatic potential) cells, proliferation was inhibited by ATL‐1 for up to 72 h. ATL‐1 also disrupted stemness maintenance in both LoVo‐CSCs (Figure 1D) and HT29‐CSCs (Figure 1E), as revealed by the decreased protein expression of stemness indicators (OCT‐4, SOX‐9, and Nanog). Next, the migration and invasion of CSCs was investigated using Transwell assays. HT29‐CSCs exhibited low metastatic potential, and their limited migratory and invasive abilities resulted in unsuccessful trials of Transwell assay. For this reason, only results for LoVo‐CSCs are shown. Evidently, ATL‐1 at 200 µM significantly decreased the migration (Figure 1F) and invasion (Figure 1G) of LoVo‐CSCs. These results are complemented by flow cytometry observations showing that the administration of ATL‐1 induced remarkable apoptosis in CSCs compared to that in nontreated cells (Figure 1H). Corresponding flow cytometric profiles of cell cycle progression in CSCs are provided in Figure S2.

### 3.2 Relationship between miR‐200c and the metastatic potential of colorectal CSCs

**FIGURE 2:**
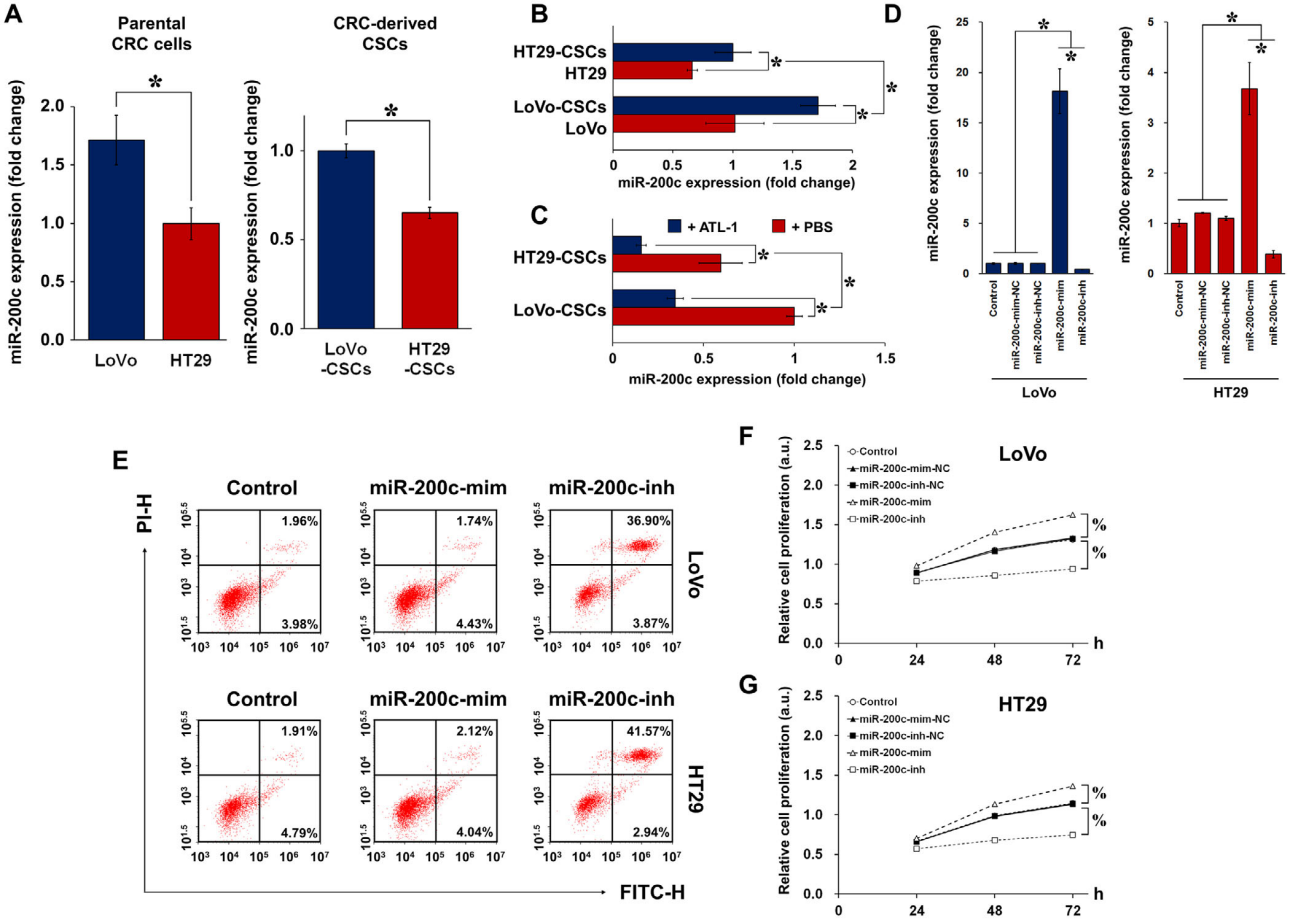
miR‐200c expression in parental CRC cells and colorectal CSCs. A, Relative basal expression of miR‐200c in LoVo cells (high metastatic potential) and HT29 cells (low metastatic potential), as well as isolated CSCs (LoVo‐CSCs and HT29‐CSCs), demonstrates a possible relationship between miR‐200c and metastatic property. B, miR‐200c expression in LoVo and HT29 cells relative to that of their corresponding CSCs. The same number of colorectal CSCs clearly exhibited higher miR‐200c expression than did CRC cells. C, miR‐200c expression was attenuated by ATL‐1 at 200 μM in both LoVo‐CSCs and HT29‐CSCs. D, Transfection efficiency of miR‐200c mimics (miR‐200c‐mim), inhibitors (miR‐200c‐inh), and their corresponding negative controls (miR‐200c‐mim‐NC and miR‐200c‐inh‐NC) in LoVo and HT29 cells. miR‐200c‐mim and miR‐200c‐inh, respectively, induced significant upregulation and downregulation of miR‐200c in LoVo and HT29 cells. E, The percentage of apoptotic LoVo and HT29 cells was increased by miR‐200c‐inh but remained unchanged in the case of miR‐200c‐mim. Relative proliferation of (F) LoVo and (G) HT29 cells subjected to transfection of miR‐200c mimics or inhibitors (or their corresponding NC). miR‐200c‐mim and miR‐200c‐inh, respectively, enhanced and inhibited the proliferation of both types of parental CRC cells. The data represent the mean ± SD of three independent technical replicates (*t*‐test or ANOVA); ^*^ *P* < .05; % *P* < .05 at 72 h

To elucidate whether there is a correlation between miR‐200c and the metastatic potential of CRC cells and colorectal CSCs, we detected the expression of miR‐200c in LoVo and HT29 cells. We anticipated that the highly metastatic LoVo cells would exhibit higher expression of miR‐200c than HT29 cells, and the results confirmed our speculation (Figure 2A). The same trend was observed in the CSCs derived from the respective CRC cells. We then proceeded to compare the expression of miR‐200c in colorectal CSCs with that in the CRC cell line from which they were isolated. Comparing the same number of LoVo (or HT29) cells and LoVo‐CSCs (or HT29‐CSCs), the CSCs showed a clear increase in miR‐200c expression (Figure 2B). Administration of 200 μM ATL‐1 to CSCs significantly attenuated the expression of miR‐200c compared to that in nontreated CSCs (Figure 2C). From these results, it is speculated that the high metastatic potential of LoVo cells may be associated with the expression of miR‐200c in the CSC subpopulation.

**FIGURE 3:**
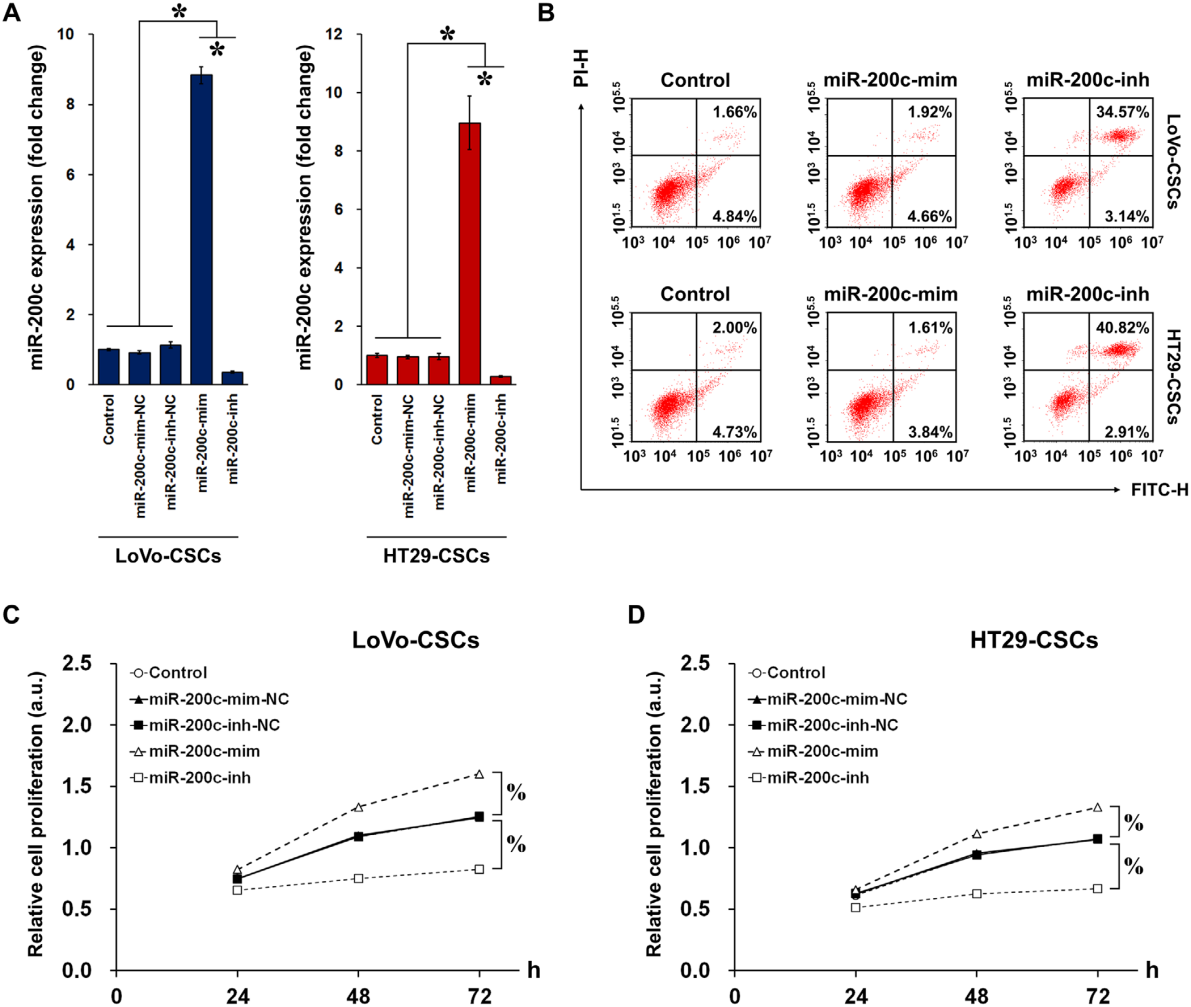
Transfection of miR‐200c mimics/inhibitors in colorectal CSCs. A, Transfection efficiency of miR‐200c mimics (miR‐200c‐mim), inhibitors (miR‐200c‐inh), and their corresponding negative controls (miR‐200c‐mim‐NC and miR‐200c‐inh‐NC) in LoVo‐CSCs and HT29‐CSCs. miR‐200c‐mim and miR‐200c‐inh, respectively, induced significant upregulation and downregulation of miR‐200c in both LoVo‐CSCs and HT29‐CSCs. B, The percentage of apoptotic LoVo‐CSCs and HT29‐CSCs was increased by miR‐200c‐inh but remained unchanged in the case of miR‐200c‐mim. Relative proliferation of (C) LoVo‐CSCs and (D) HT29‐CSCs subjected to transfection of miR‐200c mimics or inhibitors (or their corresponding NC). miR‐200c‐mim and miR‐200c‐inh, respectively, enhanced and inhibited the proliferation of both types of colorectal CSCs. The data represent the mean ± SD of three independent technical replicates (ANOVA); ^*^ *P* < .05; % *P* < .05 at 72 h

We further investigated the role of miR‐200c in regulating CRC cell behavior, in particular proliferation and apoptosis. LoVo and HT29 cells were treated with miR‐200c mimics (miR‐200c‐mim) or inhibitors (miR‐200c‐inh). The transfection efficiency was excellent in LoVo cells, wherein miR‐200c‐mim and miR‐200c‐inh significantly upregulated and downregulated the expression of miR‐200c, respectively. However, in HT29 cells, the inhibitor demonstrated high efficiency, whereas the efficiency of the mimic was suboptimal (Figure 2D). After mimic or inhibitor treatment, CRC apoptosis and cell proliferation were examined using flow cytometry and MTT assay, respectively. In terms of apoptosis, miR‐200c‐inh induced an increase in the percentage of apoptotic LoVo and HT29 cells (Figure 2E). Corresponding flow cytometric analysis of cell cycle progression is demonstrated in Figure S3. In addition, in both LoVo (Figure 2F) and HT29 (Figure 2G) cells, proliferation was enhanced by miR‐200c‐mim but suppressed by miR‐200c‐inh. Together with the previous results, these findings suggest that miR‐200c confers CRC cells with metastatic traits by promoting cell proliferation and inhibiting apoptosis. Subsequently, we investigated whether miR‐200c overexpression and interference exert similar effects in colorectal CSCs as they did in CRC cells. LoVo‐CSCs and HT29‐CSCs were transfected with miR‐200cmimics or inhibitors, and the transfection efficiency was validated (Figure 3A). Similar to the CRC cells, flow cytometric analysis showed a marked increase in apoptosis in both types of CSCs transfected with miR‐200c‐inh (Figure 3B). Corresponding flow cytometric analysis of cell cycle progression in transfected CSCs is demonstrated in Figure S4. This was supported by the MTT assay, which revealed that the proliferation of both types of CSCs was enhanced by miR‐200c‐mim but inhibited by miR‐200c‐inh over a period of 72 h (Figures 3C and 3D). Collectively, the results demonstrate that transfection of miR‐200c mimics and inhibitors had a direct effect on CSC behavior. Thus, the effects of miR‐200c observed previously in CRC cells may be a result of changes in the properties of the colorectal CSC subpopulation found within.

### 3.3 Characterization of EVs derived from miR‐200c‐transfected colorectal CSCs

**FIGURE 4:**
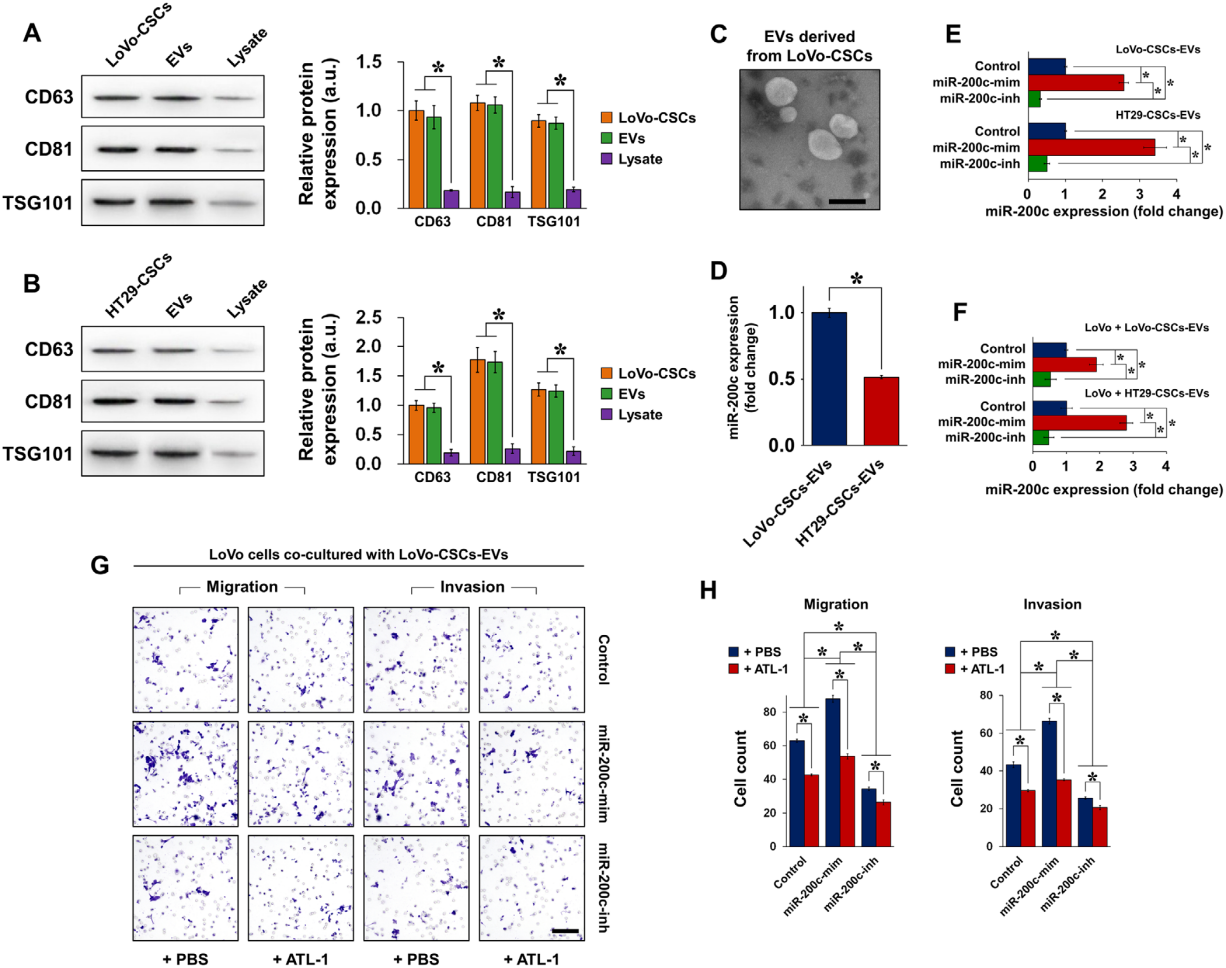
Isolation and characterization of CSC‐derived EVs as a carrier of miR‐200c. A, Expression of exosomal markers CD63, CD81, and TSG101 was detected in LoVo‐CSCs, EVs isolated from LoVo‐CSCs, and cell lysates after isolation. B, Expression of exosomal markers CD63, CD81, and TSG101 was detected in HT29‐CSCs, EVs isolated from HT29‐CSCs, and cell lysates after isolation. In both cases, the lysates of the CSCs showed negligible expression of these markers compared to that in CSCs and CSC‐derived EVs. C, Transmission electron microscopy of EVs derived from LoVo‐CSCs (scale bar = 200 nm). D, Comparison of the relative basal expression of miR‐200c in EVs derived from nontransfected LoVo‐CSCs and HT29‐CSCs. LoVo‐CSCs‐EVs expressed higher levels of miR‐200c than did HT29‐CSCs‐EVs. E, Expression of miR‐200c in EVs derived from LoVo‐CSCs or HT29‐CSCs that were first transfected with miR‐200c mimics (miR‐200cmim) or inhibitors (miR‐200c‐inh). EVs derived from nontransfected CSCs are denoted as Control. miR‐200c‐mim and miR‐200c‐inh, respectively, induced significant upregulation and downregulation of miR‐200c in both LoVo‐CSCs‐EVs and HT29‐CSCs‐EVs relative to Control levels. F, Expression of miR‐200c in LoVo cells co‐cultured with LoVo‐CSCs‐EVs and HT29‐CSCs‐EVs (CSCs were subjected to various transfections). Transfection of CSCs with miR‐200c‐mim and miR‐200c‐inh resulted in the secretion of EVs that, respectively, induced significant upregulation and downregulation of miR‐200c in LoVo cells relative to Control levels. G, Transwell assay of the migration and invasion of LoVo cells co‐cultured with LoVo‐CSCs‐EVs (CSCs were subjected to various transfections), with or without ATL‐1 administration at 200 μM. Relative to Control levels, migration and invasion were enhanced by EVs derived from miR‐200c‐mim‐transfected CSCs, but suppressed by those derived from miR‐200c‐inh‐transfected CSCs. In all cases, ATL‐1 reduced the degree of migration and invasion. Scale bar = 50 μm. H, Quantification of the data in (F) by cell count. The data represent the mean ± SD of three independent technical replicates (*t*‐test or ANOVA) ^*^ *P* < .05

We then explored whether the properties conferred by miR‐200c can be horizontally transferred between cells via a carrier agent. To do this, we isolated EVs from LoVo‐CSCs and HT29‐CSCs (LoVo‐CSCs‐EVs and HT29‐CSCs‐EVs, respectively). Western blot of exosomal markers CD63, CD81, and TSG101^20^ confirmed successful EV isolation, as these markers were exclusively expressed in EVs and were almost nonexistent in the lysates of CSCs from which the EVs were derived (Figures 4A and 4B). EVs were also identified by TEM (Figure 4C). Next, we examined whether the CSC‐derived EVs acted as a carrier for miRNAs, in particular miR‐200c. We first confirmed that the basal level of miR‐200c was lower in HT29‐CSCs‐EVs than that in LoVo‐CSCs‐EVs (Figure 4D). Then, CSCs isolated from LoVo or HT29 cells were transfected with miR‐200c mimics or inhibitors as described, and EVs were isolated from the transfected CSCs. EVs from nontransfected CSCs acted as controls. We observed that miR‐200c‐mim‐transfected LoVo‐CSCs and HT29‐CSCs produced EVs that exhibited significantly elevated miR‐200c expression than did the nontransfected CSCs. Contrarily, transfection of miR‐200c‐inh in CSCs resulted in the isolation of EVs with attenuated miR‐200c expression compared to that in control EVs (Figure 4E). To determine whether EVs carrying miR‐200c mediated the horizontal transfer of metastatic traits, we cultured LoVo cells with EVs derived from either LoVo‐CSCs or HT29‐CSCs transfected with miR‐200c mimics or inhibitors. LoVo cells showed higher expression of miR‐200c when miR‐200c‐overexpressing EVs were added, whereas correspondingly, EVs from miR‐200c‐inh‐transfected CSCs led to lower miR‐200c expression (Figure 4F). The migratory and invasive capabilities of the cultured LoVo cells were then assessed using a Transwell assay, with EVs from nontransfected CSCs used as controls. In the case of both LoVo‐CSCs and HT29‐CSCs, the transfection of miR‐200c‐mim in the CSCs resulted in EVs that greatly enhanced the migratory and invasive capabilities of LoVo cells. Interference of miR‐200c, on the other hand, produced CSC‐derived EVs that limited the migration and invasion of LoVo cells (Figures 4G and 4H). We also examined whether 200 μM ATL‐1 had an inhibitory effect on migration and invasion, as it did on cell proliferation demonstrated previously. As anticipated, ATL‐1 impaired the migration and invasion of LoVo cells in the presence of EVs, as confirmed by the significant decrease in cell count in the case of transfected and nontransfected CSCs (Figures 4G and 4H).

### 3.4 Co‐culture of LoVo and HT29 cells with CSC‐derived EVs

**FIGURE 5:**
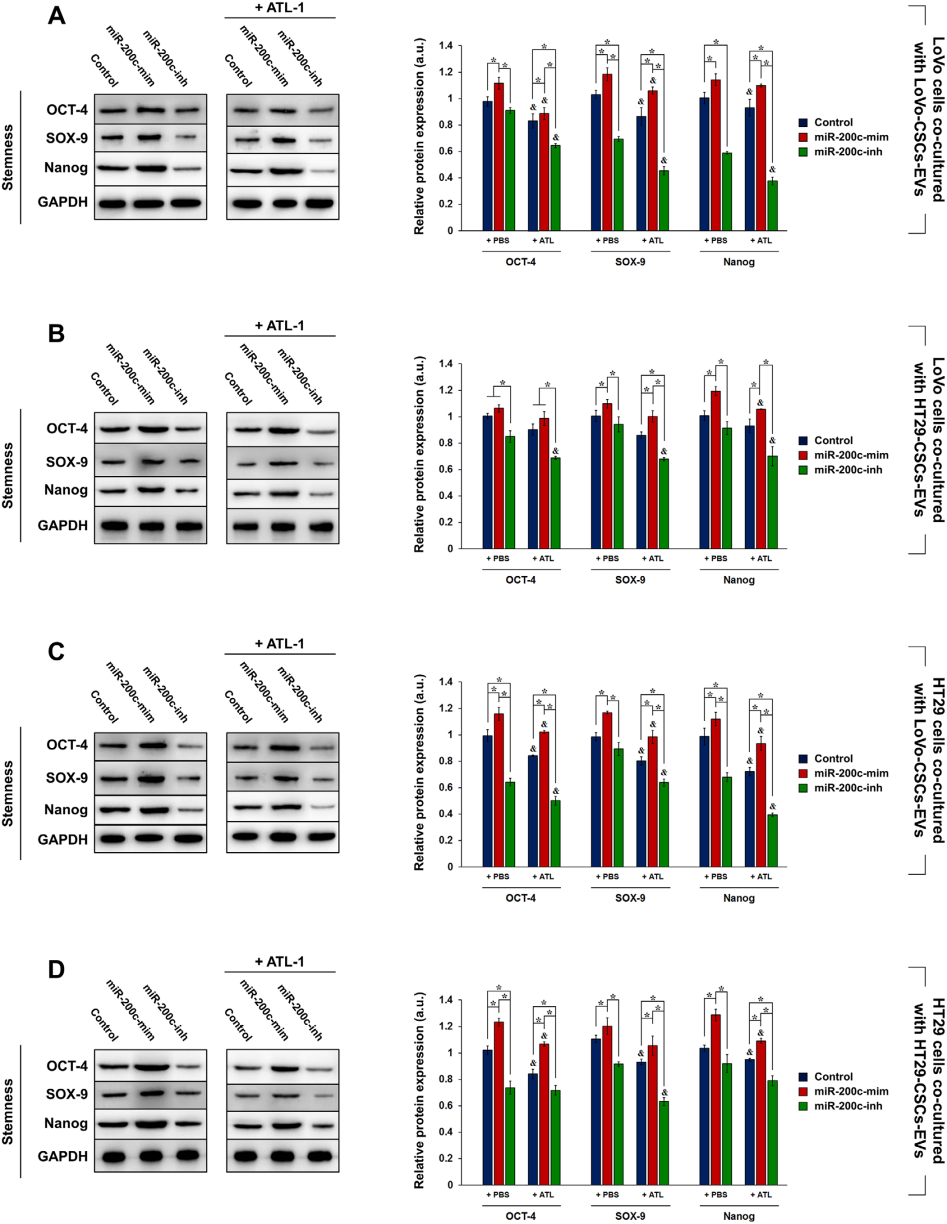
Effect of EV co‐culture on stemness maintenance in LoVo and HT29 cells. LoVo‐CSCs and HT29‐CSCs were first transfected with miR‐200c mimics (miR‐200c‐mim) or inhibitors (miR‐200c‐inh). LoVo cells were co‐cultured for 48 h with EVs isolated from nontransfected (Control) or transfected (A) LoVo‐CSCs or (B) HT29‐CSCs, with or without ATL‐1 administration at 200 μM. Similarly, HT29 cells were co‐cultured for 48 h with EVs isolated from nontransfected (Control) or transfected (C) LoVo‐CSCs or (D) HT29‐CSCs, with or without ATL‐1 administration at 200 μM. Western blot and quantification of stemness maintenance markers OCT‐4, SOX‐9, and Nanog relative to GAPDH were carried out. Overexpression of miR‐200c in EVs enhanced the stem‐like properties of LoVo and HT29 cells via horizontal transfer, whereas miR‐200c interference suppressed these traits. The data represent the mean ± SD of three independent technical replicates (ANOVA); ^*^ *P* < .05; *P* < .05 versus the same group with PBS only (+PBS)

We were interested in the specific effect of CSC‐derived EVs on the stemness maintenance of non‐CSCs and the involvement of miR‐200c therein. LoVo or HT29 cells were cultured with EVs derived from LoVo‐CSCs or HT29‐CSCs that were not transfected (Control) or transfected with miR‐200c mimics or inhibitors, and the expression of stemness markers OCT‐4, SOX‐9, and Nanog was evaluated. Compared to control EVs (from nontransfected CSCs), those isolated from miR‐200c‐mim‐transfected LoVo‐CSCs and HT29‐CSCs induced stronger expression of stemness markers, whereas EVs isolated from miR‐200c‐inh‐transfected CSCs suppressed their expression. This was the general trend observed in LoVo cells co‐cultured with LoVo‐CSCs‐EVs (Figure 5A) or HT29‐CSCs‐EVs (Figure 5B), as well as in HT29 cells co‐cultured with LoVo‐CSCs‐EVs (Figure 5C) or HT29‐CSCs‐EVs (Figure 5D). Additionally, we observed that the incorporation of 200 μM ATL‐1 in the co‐culture of CRC cells and EVs had an inhibitory effect on stemness, as demonstrated by the decrease in OCT‐4, SOX‐9, and Nanog in all cases.

We also investigated whether miR‐200c carried by isolated EVs affected the activation of the PI3K/Akt/mTOR signaling pathway in CRC cells. The same co‐cultures were performed and the expression of phosphorylated PI3K, Akt, and mTOR was detected relative to the respective total protein content. Similar to stemness maintenance, PI3K/Akt/mTOR activation was promoted in CRC cells co‐cultured with EVs from miR‐200c‐mim‐transfected CSCs but decreased by miR‐200c interference.

**FIGURE 6:**
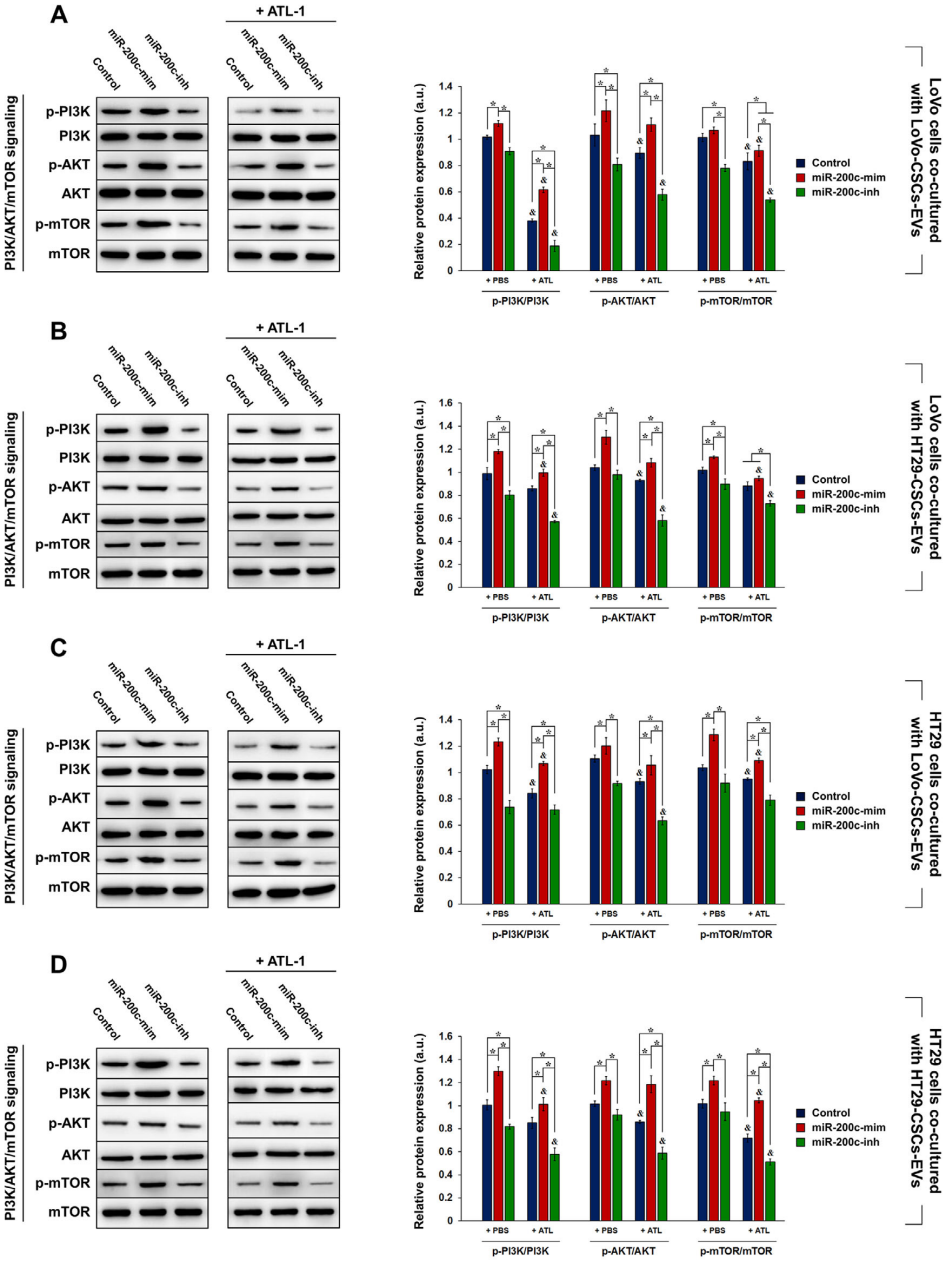
Effect of EV co‐culture on PI3K/AKT/mTOR signaling in LoVo and HT29 cells. LoVo‐CSCs and HT29‐CSCs were first transfected with miR‐200c mimics (miR‐200c‐mim) or inhibitors (miR‐200c‐inh). LoVo cells were co‐cultured for 48 h with EVs isolated from nontransfected (Control) or transfected (A) LoVo‐CSCs or (B) HT29‐CSCs, with or without ATL‐1 administration at 200 μM. Similarly, HT29 cells were co‐cultured for 48 h with EVs isolated from nontransfected (Control) or transfected (C) LoVo‐CSCs or (D) HT29‐CSCs, with or without ATL‐1 administration at 200 μM. Western blot and quantification of the phosphorylation levels of PI3K, AKT, and mTOR relative to the total level of the corresponding protein were carried out. Overexpression of miR‐200c in EVs enhanced the activation of the PI3K/AKT/mTOR signaling pathway in LoVo and HT29 cells via horizontal transfer, whereas miR‐200c interference suppressed these traits. The data represent the mean ± SD of three independent technical replicates (ANOVA); ^*^ *P* < .05; *P* < .05 versus the same group with PBS only (+PBS)

This was the case for LoVo cells co‐cultured with LoVo‐CSCs‐EVs (Figure 6A) or HT29‐CSCs‐EVs (Figure 6B), as well as in HT29 cells co‐cultured with LoVo‐CSCs‐EVs (Figure 6C) or HT29‐CSCs‐EVs (Figure 6D). The addition of 200 μM ATL‐1 induced a decrease in the phosphorylation of the abovementioned proteins, signifying that PI3K/Akt/mTOR activation was inhibited. The results suggest that EVs derived from CSCs carried different amounts of miR‐200c depending on the transfection. The EVs in turn mediated horizontal transfer of the miR‐200c cargo into LoVo or HT29 cells and conferred the cells with miR‐200c‐induced traits. Thus, the CSC‐derived EVs were able to modulate stemness maintenance and PI3K/Akt/mTOR activation to varying degrees in CRC cells.

### 3.5 Effect of ATL‐1 on uptake of CSC‐derived EVs by LoVo and HT29 cells

**FIGURE 7:**
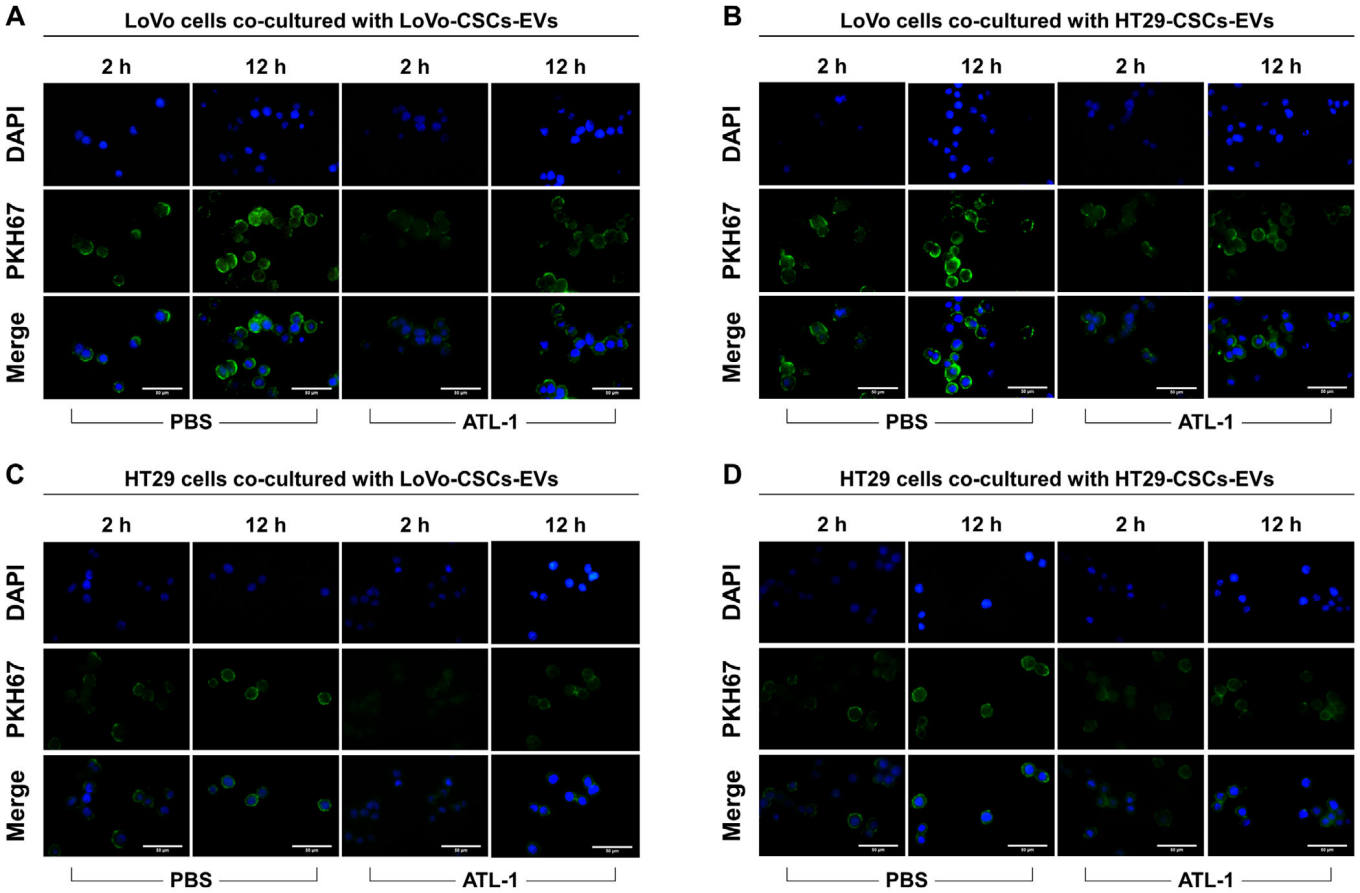
EV uptake in parental CRC cells in the presence or absence of ATL‐1. EV uptake by parental CRC cells (non‐CSCs) was evaluated by PKH67 staining. Positive green fluorescence indicates PKH67 signal, representing successful EV uptake. Blue staining represents DAPI (nuclei). EVs were labeled with PKH67 and imaging was performed after 2 and 12 h in LoVo cells co‐cultured with (A) LoVo‐CSCs‐EVs or (B) HT29‐CSCs‐EVs, or in HT29 cells co‐cultured with (C) LoVo‐CSCs‐EVs or (D) HT29‐CSCs‐EVs, in the presence or absence (PBS) of ATL‐1 at 200 μM. The CSCs from which EVs were derived were not transfected. In all cases, the stronger PKH67 fluorescence signal at 12 h compared to that at 2 h indicated the continuous uptake and accumulation of EVs by CRC cells. Relative to PBS, ATL‐1 induced lower EV uptake in all groups at both time points, as demonstrated by the weaker green fluorescence intensity. EV uptake was generally more efficient in LoVo cells than that in HT29 cells. Scale bar = 50 μm

We have shown that ATL‐1 exerted inhibitory effects on the maintenance of stemness in colorectal CSCs, as well as their growth and survival. Concurrently, we demonstrated that ATL‐1 inhibited the expression of miR‐200c in CSCs. Having shown the effect of miR‐200c‐containing EVs derived from transfected CSCs, we hypothesized that ATL‐1 could also affect the interaction between CRC cells and CSC‐derived EVs. To verify this, we added 200 μM ATL‐1 to the co‐culture of LoVo or HT29 cells with CSC‐derived EVs and evaluated the effect of ATL‐1 on EV uptake by CRC cells. PKH67 staining is a typical method of assessing the efficiency of EV uptake. As demonstrated in Figure 7, EVs that were endocytosed by LoVo or HT29 cells are identified by green staining. After 2 and 12 h of incubation, images of PKH67‐stained cells were acquired (images of cells cultured in the absence of EVs or cultured with nonlabeled EVs are shown in Figure S5). Without ATL‐1 (incubation with PBS), LoVo cells showed areas of positive staining at 2 h, and the fluorescence signal of PKH67 was remarkably enhanced at 12 h, indicating that EVs (from both LoVo‐CSCs and HT29‐CSCs) were endocytosed efficiently. In the presence of ATL‐1, the fluorescence intensity of PKH67 declined sharply at both 2 and 12 h compared to that in cells cultured in the absence of ATL‐1. The same phenomenon was observed with the uptake of both types of EVs, indicating that ATL‐1 impaired the ability of LoVo cells to endocytosed EVs (Figures 7A and 7B). On the other hand, although the fluorescence intensity of PKH67 seemed to have increased from 2 to 12 h in HT29 after co‐culture with EVs (no ATL‐1), the difference was not as apparent as that in LoVo cells. This indicates that the EV uptake ability of HT29 was not as high as that of LoVo cells. However, the addition of ATL‐1 still prevented HT29 cells from absorbing EVs, as demonstrated by the decrease in positive PKH67 signal (Figures 7C and 7D).

Taken together, ATL‐1 seemed to prevent CSC‐derived EVs from entering CRC cells, thereby restricting the horizontal transfer of miR‐200c contained within the EVs. We propose that this may be one of the mechanisms through which ATL‐1 suppresses CRC metastasis. Specifically, ATL‐1 impaired the pro‐metastatic functions of miR‐200c from being exerted via transfer to non‐CSCs by EVs.

## 4 DISCUSSION

**FIGURE 8:**
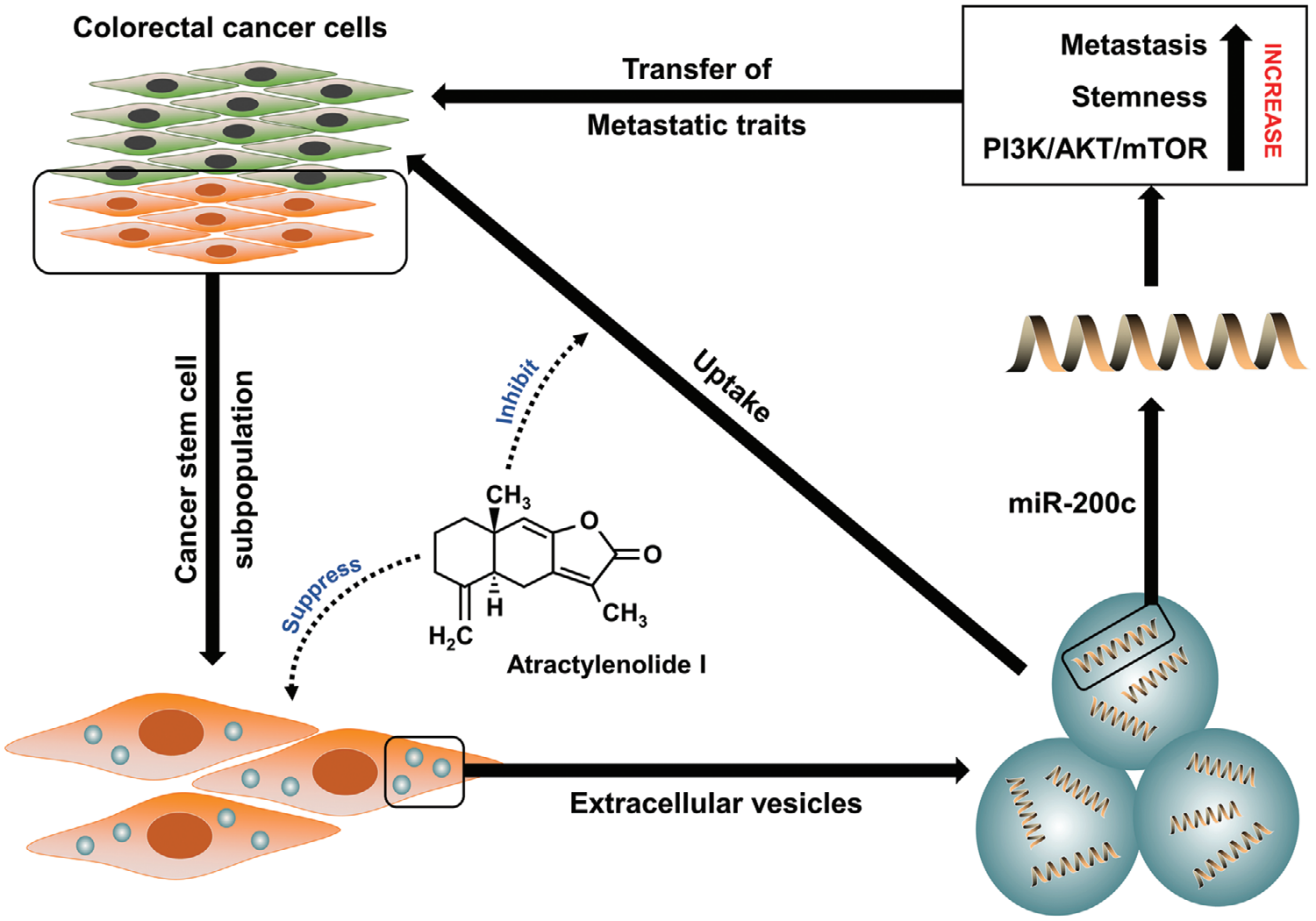
Schematic outline of the mechanism revealed in this study. A key factor that contributes to colorectal tumor metastasis is the production of a large number of CSCs in the tumor microenvironment. These CSCs secrete EVs that act as carrier vehicles to transport miR‐200c, which regulates stemness maintenance in CRC cells and promotes CRC metastasis. Horizontal transfer of miR‐200c‐mediated properties is facilitated via the co‐culture of CRC cells and miR‐200c‐containing EVs, and the uptake of EVs by CRC cells results in increased metastatic potential, enhanced stem‐like traits, and activation of PI3K/AKT/mTOR signaling. ATL‐1 suppresses the activity of CSCs while inhibiting the uptake of EVs by CRC cells to prevent metastasis

The findings of our study revealed that miR‐200c‐containing EVs isolated from colorectal CSCs contribute to the metastatic ability of CRC cells (non‐CSCs). The transfer of metastatic traits is manifested by altered CRC cell behavior, increased stemness, and activation of the PI3K/AKT/mTOR pathway via the co‐culture of CRC cells and CSC‐derived EVs (Figure 8). Herein, we showed that ATL‐1, a natural crude component of baizhu capable of suppressing cancer cell growth and self‐renewal, not only downregulated the expression of miR‐200c in colorectal CSCs, but also seemed to inhibit the uptake of miR‐200c‐containing EVs by CRC cells to prevent the conferral of metastatic properties.

To our knowledge, this is the first study that has taken a close look at the interaction between ATL‐1 and CSC‐derived EVs in the context of miR‐mediated CRC metastasis. Aberrant expression of miRs has been linked to the development of various diseases, including cardiovascular conditions,^21^ neurological disorders,^22^ and cancer.^23^ Evidence has suggested that the specific role of miR‐200c in cancer progression and metastasis could be pleiotropic or context dependent. Overexpression of miR‐200c has been shown to be associated with enhanced metastatic colonization in breast cancer.^24^ In a clinical study of CRC by Toiyama et al, serum miR‐200c was identified as a marker of tumor metastasis and poor prognosis.^25^ However, others have reported that miR‐200c may exert tumor‐suppressing functions via the regulation of CRC stemness and metastasis.^6^, ^26^ The apparent dual roles of miR‐200c are in agreement with a common phenomenon reported in many studies, wherein a single miR can act as either an oncomiR or a tumor suppressor. This could be due to the different genetic origins of the cells and/or animals used in the studies, which may affect tumorigenic mechanisms.^27^ In addition, other physiological factors such as hormonal regulation may play unexpected roles in miR‐associated metastatic progression.^28^

Stemness maintenance plays a critical role in regulating the biological properties of malignant tumors and is strongly linked to metastasis.^29^, ^30^ CSCs undergo self‐renewal and differentiation, similar to normal stem cells, and these processes may induce relapse, drug resistance, and tumor metastasis,^31^, ^32^ rendering conventional anticancer therapies ineffective. In the current research, we evaluated the expression of miR‐200c in two CRC cell lines with distinct differences in metastatic potential. LoVo cells are highly metastatic, whereas HT29 cells lack metastatic capabilities.^33^, ^34^, ^35^ We confirmed the connection between miR‐200c and metastasis by identifying that its expression was elevated in LoVo cells but weak in HT29 cells. CSCs isolated from the two CRC cell lines also showed elevated expression of miR‐200c compared to that in native CRC cells. The selective removal of miR‐200c from CSCs effectively disrupted biological behavior by inhibiting cell proliferation and inducing apoptosis. At the same time, we showed that altering the expression of miR‐200c in CSCs not only affected the behavior of the CSCs themselves, but also resulted in corresponding changes in the content of secreted EVs, characterized as CSC‐derived EVs.

EVs act as agents that facilitate cell‐cell communication and signal transduction in the tumor environment by delivering and releasing their contents.^36^ The intercellular exchange of exosomal cargo has been demonstrated to alter the phenotype of CRC cells. For example, exosomes (a type of EV) containing p‐STAT3 derived from 5‐fluorouracil‐resistant CRC cells conferred the properties of drug resistance to drug‐sensitive cells.^37^ In terms of metastasis, hypoxic CRC cells secreted exosomes carrying Wnt4, and these exosomes mediated the transfer of pro‐metastatic traits to normoxic CRC cells.^38^ By studying EVs, we partly clarified the mechanism through which metastasizing cells confer the ability to metastasize to neighboring nonmetastasizing cells. We suggest that CSC‐derived EVs are a key component of this metastatic transfer process, as they act as a carrier for the delivery of oncogenic miR‐200c to non‐CSCs that may otherwise refrain from metastasizing. Thus, the establishment of therapeutic compounds that can combat this metastatic transfer from happening may aid in preventing the exacerbation of CRC via recurrence or distant metastasis. Herein, we demonstrated the utility of ATL‐1 for this purpose. Research has shown the unique therapeutic potential of ATLs against cancers such as melanoma,^39^ lung cancer,^40^ and ovarian cancer.^41^ In particular, Zhang et al revealed that ATL‐II, another member of the ATL family with similar structural characteristics as ATL‐1, improved the chemotherapeutic efficacy against CRC by suppressing the viability and proliferation of CRC cells. More importantly, this ATL increased the chemosensitivity of CRC cells to conventional chemotherapeutic drugs including 5‐fluorouracil, mitomycin, cisplatin, and Adriamycin, and this was achieved by modulating the lncRNA XIST/miR‐30a‐3p/ROR 1 axis.^42^

Through our investigation, we found that the function of ATL‐1 is twofold. On one hand, ATL‐1 acted directly on CSCs by suppressing stemness maintenance and promoting CSC apoptosis. On the other hand, ATL‐1 had an inhibitory effect on EV uptake. When CRC cells were cultured with CSC‐derived EVs, they gained metastatic activity through the horizontal transfer of miR‐200c. Thus, the decreased uptake of EVs by CRC cells indicates that the transfer of miR‐200c‐associated traits is halted, further supporting the antimetastatic role of ATL‐1. Moreover, we revealed the involvement of the PI3K/AKT/mTOR signaling pathway in the regulation of EV‐mediated metastatic activity. The PI3K/AKT/mTOR pathway has been critically implicated in the development of CRC.^43^, ^44^ The overexpression of miR‐200c, which was presumed to be oncogenic in our research, effectively promoted the activation of PI3K/AKT/mTOR signaling, contributing to CRC progression. Furthermore, studies have suggested that the antitumor effect ATL‐1 is dependent on the inhibition of PI3K/AKT/mTOR signal activation,^17^, ^45^ which was confirmed by our results.

There are several limitations in our study. First, the CSCs used for the experiments were derived from cell lines. Ideally, CSCs should be isolated from primary cancer cells to yield more representative results of physiological scenarios, but complex ethical concerns and time‐consuming isolations processes hindered the use of primary CSCs in this study. Second, the effect of only ATL‐1 was shown in this study, and the activities of the other ATLs in baizhu remain unknown. A systematic investigation of all ATLs in comparison with baizhu should be performed to gain insights into the correlation between the crude drug and its individual components. Finally, in vivo studies in animal models and clinical data will be required to further support the translational aspects of our results.

In summary, EVs derived from colorectal CSCs carry oncogenic miR‐200c and act as a messenger to enable the horizontal transfer of metastatic properties to CRC cells, thereby enhancing their stemness maintenance and invasive capability via PI3K/AKT/mTOR activation. ATL‐1 effectively impaired the function of CSCs and blocked the transfer of miR‐200c by hindering EV uptake. Our research elucidates the process through which CRC cells acquire metastatic traits via horizontal transfer of carrier particles secreted by neighboring CSCs. The findings may be useful in the development of miR‐based anticancer schemes by targeting EV‐mediated activity.

## CONFLICT OF INTEREST

The authors declare no conflict of interest.

## Supporting information

Supporting Information.

Supporting Information.

Supporting Information.

Supporting Information.

Supporting Information.

## Data Availability

The data used in the current study are available from the corresponding author on reasonable request.
