# Supplementary figures and images for: Transfer of metastatic traits via miR‐200c in extracellular vesicles derived from colorectal cancer stem cells is inhibited by atractylenolide I
Source: Clin Transl Med. 2020 Aug 12;10(4):e139. doi: 10.1002/ctm2.139 (PMC7423185; doi:10.1002/ctm2.139)

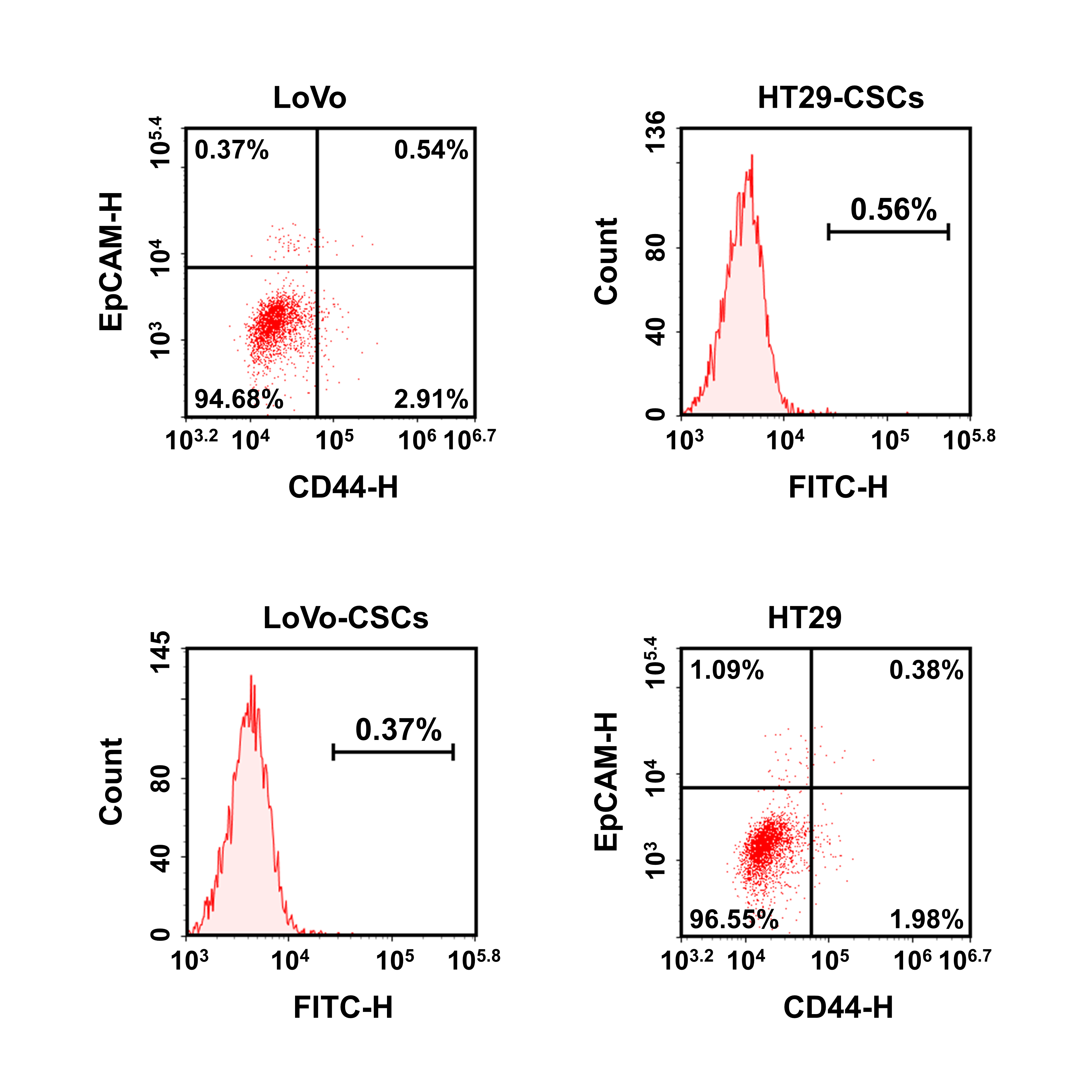

Supplement: Supplementary file 1 — Supporting Information. [file CTM2-10-e139-s001.tif]

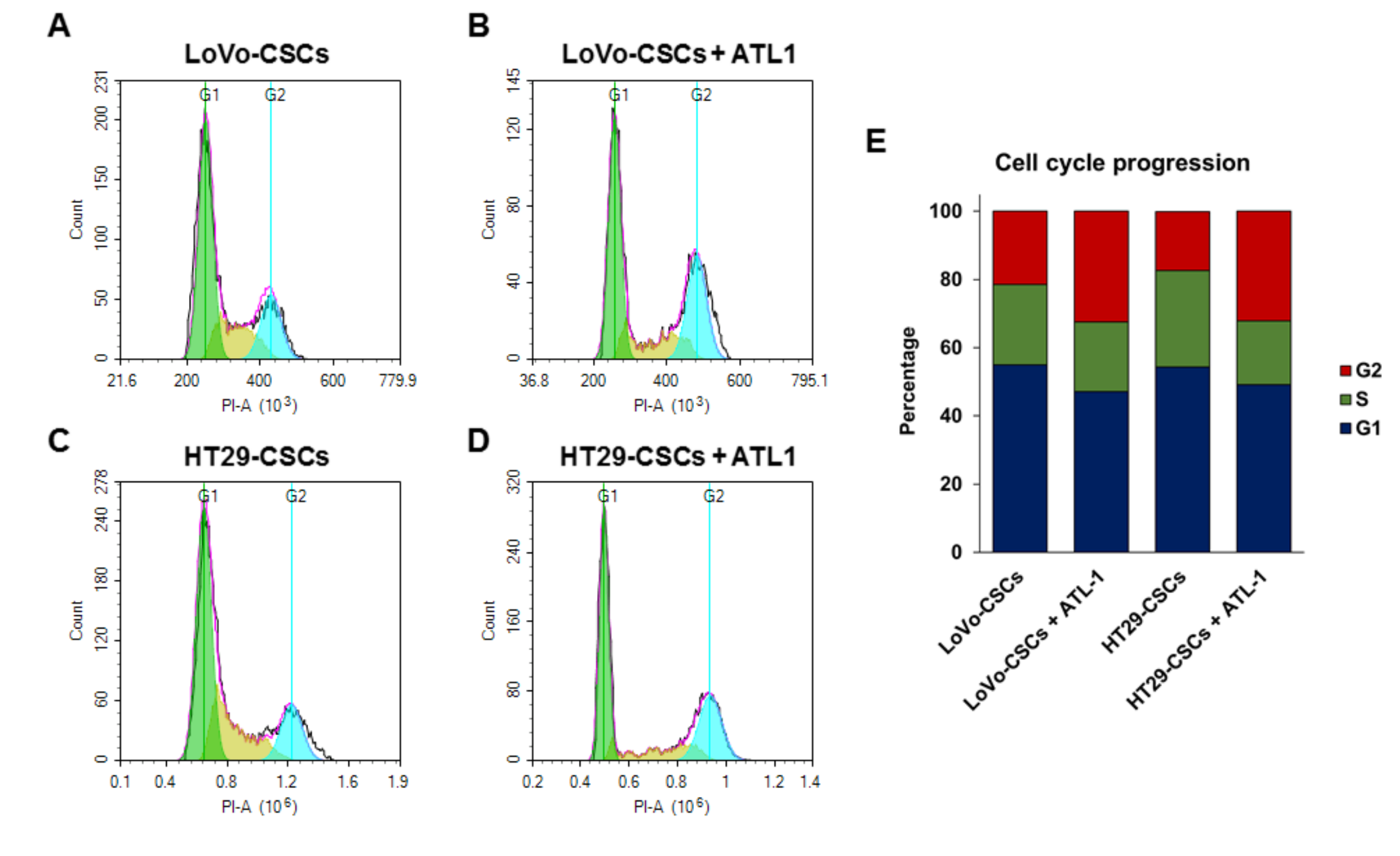

Supplement: Supplementary file 2 — Supporting Information. [file CTM2-10-e139-s002.tif]

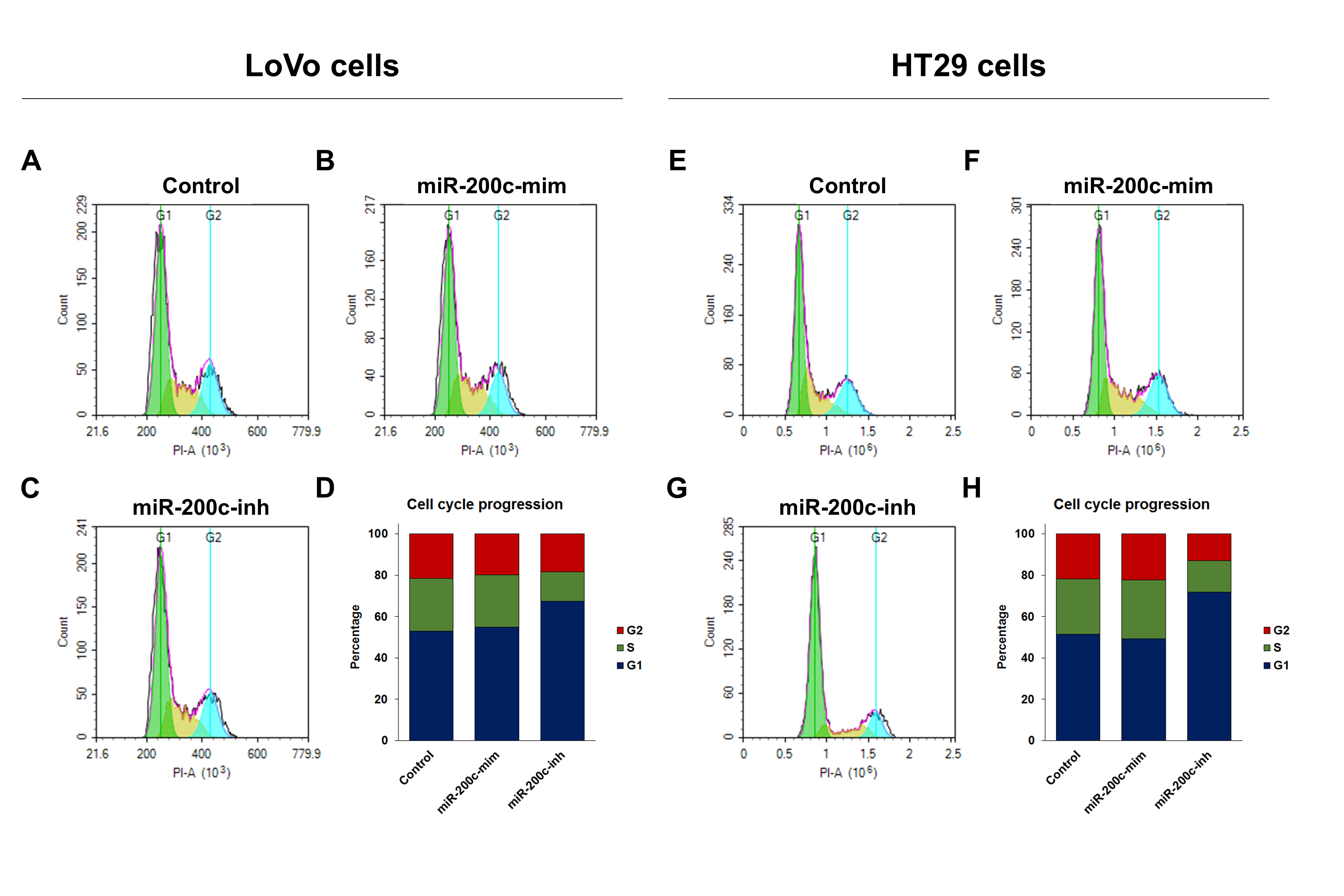

Supplement: Supplementary file 3 — Supporting Information. [file CTM2-10-e139-s003.tif]

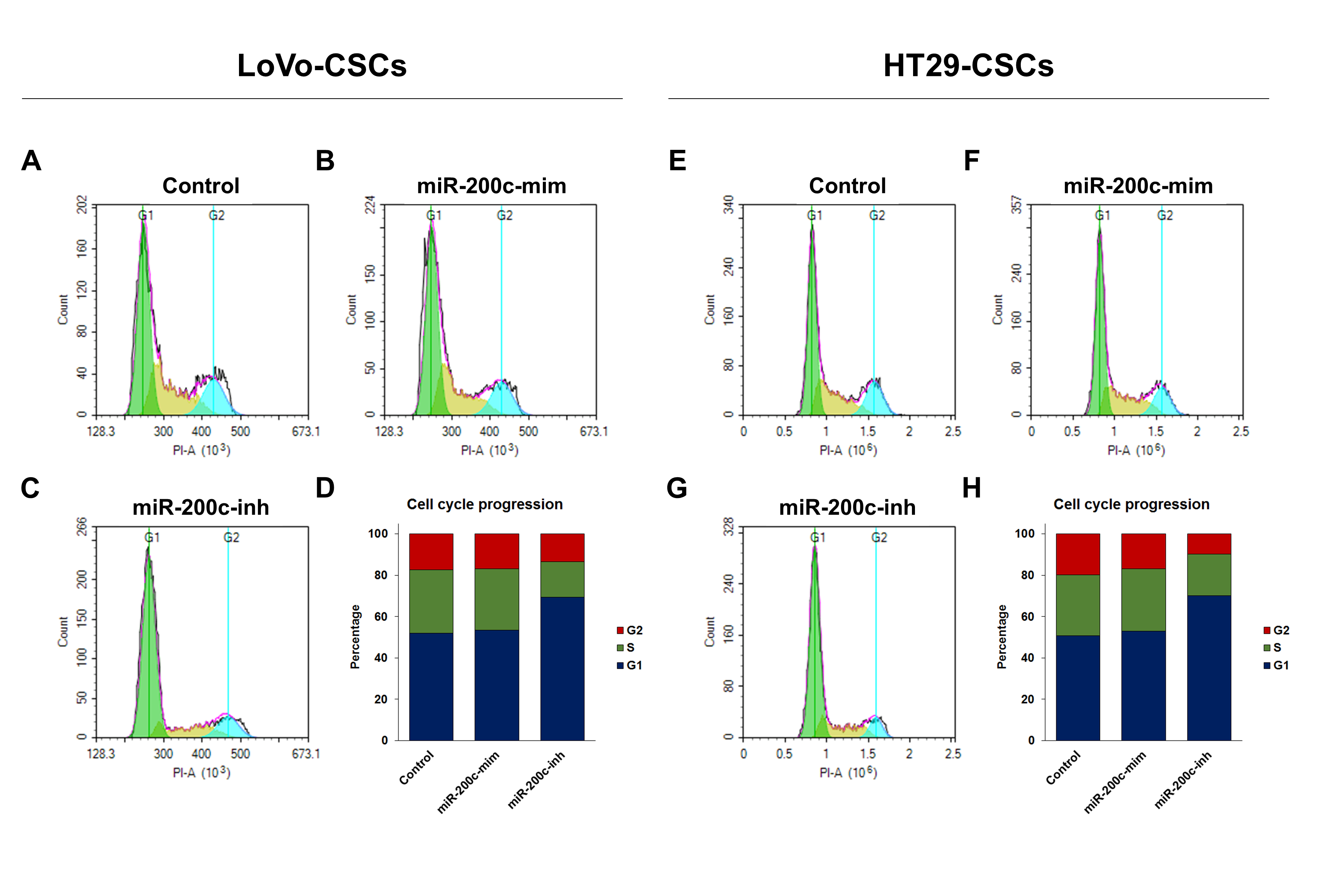

Supplement: Supplementary file 4 — Supporting Information. [file CTM2-10-e139-s004.tif]

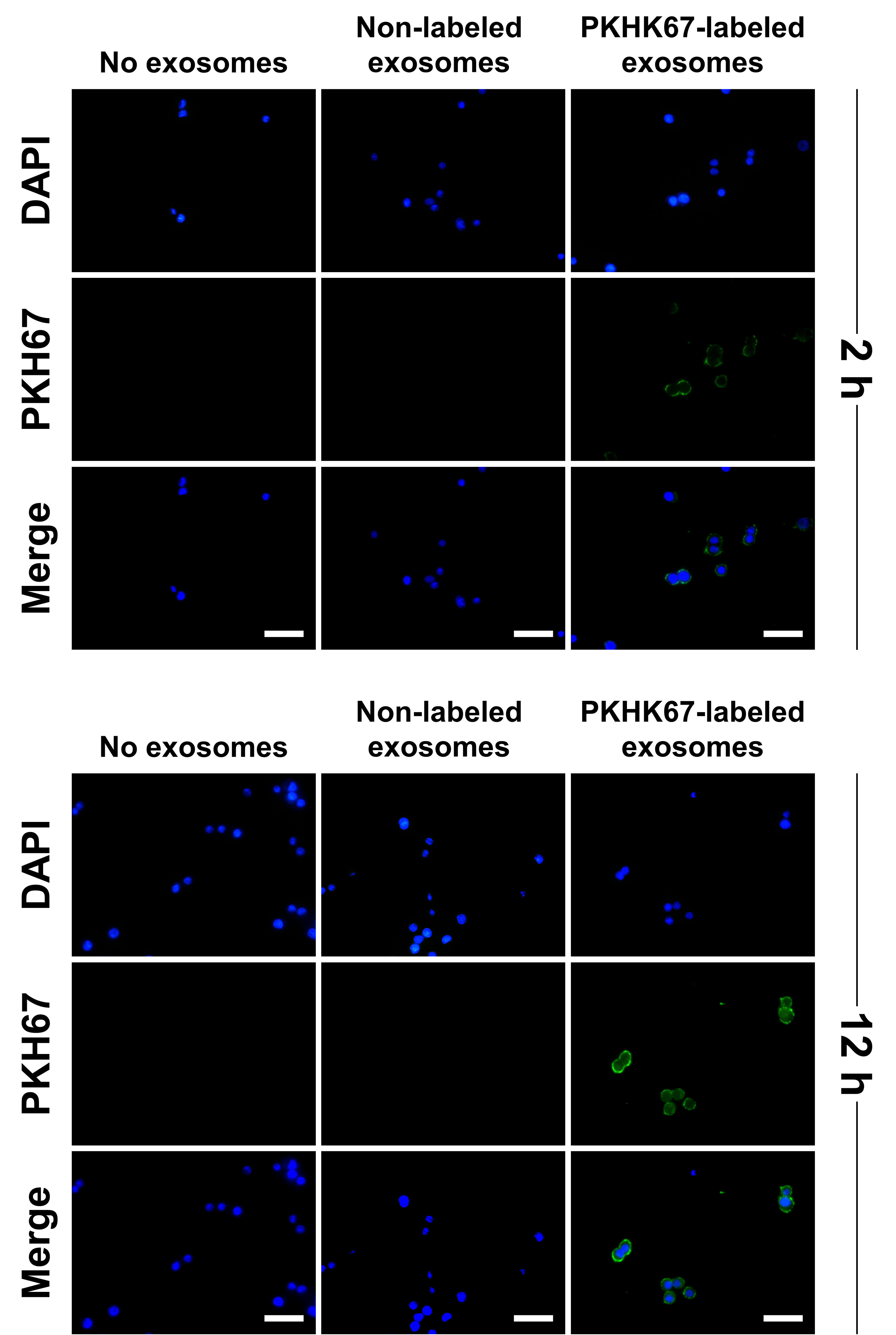

Supplement: Supplementary file 5 — Supporting Information. [file CTM2-10-e139-s005.tif]
